# Experiences of treatment decision making for young people diagnosed with depressive disorders: a qualitative study in primary care and specialist mental health settings

**DOI:** 10.1186/1471-244X-11-194

**Published:** 2011-12-12

**Authors:** Magenta B Simmons, Sarah E Hetrick, Anthony F Jorm

**Affiliations:** 1 Orygen Youth Health Research Centre, Centre for Youth Mental Health, The University of Melbourne, Locked Bag 10, Parkville 3052, Victoria, Australia; 2 headspace Centre of Excellence in Youth Mental Health, Orygen Youth Health Research Centre, Centre for Youth Mental Health, The University of Melbourne, Locked Bag 10, Parkville 3052, Victoria, Australia

## Abstract

**Background:**

Clinical guidelines advocate for the inclusion of young people experiencing depression as well as their caregivers in making decisions about their treatment. Little is known, however, about the degree to which these groups are involved, and whether they want to be. This study sought to explore the experiences and desires of young people and their caregivers in relation to being involved in treatment decision making for depressive disorders.

**Methods:**

Semi-structured interviews were carried out with ten young people and five caregivers from one primary care and one specialist mental health service about their experiences and beliefs about treatment decision making. Interviews were audio taped, transcribed verbatim and analysed using thematic analysis.

**Results:**

Experiences of involvement for clients varied and were influenced by clients themselves, clinicians and service settings. For caregivers, experiences of involvement were more homogenous. Desire for involvement varied across clients, and within clients over time; however, most clients wanted to be involved at least some of the time. Both clients and caregivers identified barriers to involvement.

**Conclusions:**

This study supports clinical guidelines that advocate for young people diagnosed with depressive disorders to be involved in treatment decision making. In order to maximise engagement, involvement in treatment decision making should be offered to all clients. Involvement should be negotiated explicitly and repeatedly, as desire for involvement may change over time. Caregiver involvement should be negotiated on an individual basis; however, all caregivers should be supported with information about mental disorders and treatment options.

## Background

Experiencing depression as a child or adolescent can have a significant impact on the social, occupational, physical and emotional functioning of the young person [1,2]. This is of concern given that by the age of 18 years one in every five people will have experienced depression [3]. It is crucial to provide optimal treatment for this age group in order to minimise the negative impact of depression on their lives [4]. Despite this need, a large proportion of those young people experiencing depression will not access treatment [5,6], and for those who do, many will experience significant delays before accessing services [7,8]. There are a number of factors that may lead to a delay in treatment, including stigma [9], negative attitudes to, and experiences of, treatment [10,11], and availability of services [12]. Once in treatment, engagement remains a considerable challenge [13].

In order to maximize the chances of successful engagement, a collaborative approach to treatment decision making has been proposed for mental health treatment in general [14] and for young people experiencing depression specifically [15]. Clinical guidelines for the treatment of depression in children and adolescents advocate for the inclusion of young people in learning about and making decisions regarding their treatment (e.g. [16]). Active involvement in treatment decision making may lead to increased satisfaction with services and therefore improve engagement and clinical outcomes [17,18].

Involvement in treatment decision making has been discussed in several ways. At a broad level, involvement can be seen to be passive (e.g. paternalistic model of care provided by a clinician, whereby the clinician shares little information with the client, and the clinician deliberates and makes the final decision about treatment); shared (e.g. the client and clinician share information with each other, both deliberate about this information and choose a treatment option together); or autonomous (e.g. the client may obtain information from the clinician but then deliberates on this information and makes a decision themselves) [19].

It is important to consider what constitutes involvement. In the above model, the conceptualization of involvement focuses on information sharing and making actual decisions. Entwistle and Watt (2006) [20] suggest a broadening of what constitutes involvement to include both 1) participation in activities related to decision making (with or without other people) and 2) the way that people think and feel about such activities and people. They describe three existing domains of involvement (communication between clients and clinicians about decision making; efforts made by the client in relation to decision making; and efforts made by the clinician in relation to decision making), and propose that two further areas should be considered in order to create a more meaningful understanding of client involvement: 1) the way in which clients view, and feel about, these first three domains as well as their role in, and contribution to, the decision-making process; and 2) consideration of the feelings of both the client and clinician about their relationship with each other.

It is also important to consider the ways in which clients may wish to be involved in the decision making process. Deber and colleagues (2007) [21] conceptualise two categories related to making decisions: 1) problem solving, which refers to a scenario where there is only one possible correct answer and is therefore not able to be influenced by client preferences; and 2) decision making, which may be based on problem solving, that can be influenced by client preferences and involves some consideration of the potential pros and cons of different scenarios (e.g. treatment options). For each of these categories, it is proposed that clients can prefer to 'keep' the responsibility themselves, 'share' responsibility with a clinician, or 'hand over' responsibility to a clinician. Combinations of these preferences then fall under categories of preference for involvement in decision making (e.g. passive, shared, autonomous). Recently, a 'shared' model of client involvement in decision making, called shared decision making, has received increased interest in both research and clinical settings.

Shared decision making (SDM) facilitated by the use of decision aids (DAs) has been well tested in areas such as breast cancer treatment decision making [e.g. [22]] and choosing whether or not to undertake screening for prostate cancer [e.g. [23]]. The most common model of SDM is that described by Charles and colleagues [24], which involves three major steps: 1) two parties (e.g. doctor and patient) provide each other with relevant information; 2) these same two parties deliberate on the decision by discussing the treatment options and preference for each option; and 3) a treatment option is selected that is consistent with patient preferences and values. This model was updated in 1999 [19] to further detail these stages of decision making (e.g. that information exchange comprises flow, direction, type and amount of information), to acknowledge that approaches taken to decision making are not necessarily static and may indeed change over time, to describe sub-categories of decision making approaches that are hybrid versions of the broader paternalistic, shared and autonomous categories, and to make the SDM model more relevant to clinical, research and educational purposes (e.g. helping clinicians to understand the different variations of involvement). Edwards and Elwyn (2006) [25] have highlighted the importance of focusing on the process of decision making (e.g. presentation of treatment options, information sharing and consideration of preferences for who should make the decision) rather than on who makes the actual decision.

DAs are evidence-based tools that provide information about the potential risks and benefits of treatment options (or other health related choices such as screening tests) and are designed to elicit patient preferences in relation to these options. In doing so, they are also designed to facilitate the exchange of information and increase the amount of information shared between the healthcare provider and consumer. The establishment of the International Patient Decision Aid Standards (IPDAS), means that the quality of DAs can now be assessed [26].

Despite enthusiasm for the application of SDM for mental disorders [e.g. [27,28]] a recent systematic review concluded that only two randomized controlled trials (RCTs) had been conducted investigating SDM for mental disorders [29]. The first study was a cluster RCT that tested an intervention that involved SDM training for physicians and a DA for adult outpatients diagnosed with depressive disorders [30]. The intervention significantly increased patient involvement and satisfaction without increasing the duration of consultations; however, the SDM intervention had no impact on level of depression severity. The second study was a single site RCT that investigated SDM with the use of a DA for inpatients diagnosed with schizophrenia [31,32]. The intervention demonstrated that SDM was feasible for this population, and significantly increased patients' knowledge about schizophrenia, uptake of psychoeducation, and feelings of involvement in consultations, again, without increasing consultation time. However, as with the first study, clinical symptom severity did not improve.

Despite there only being two RCTs testing interventions specific to SDM and the use of DAs, there have been a number of efforts in recent years that have taken a person-centered approach to interventions for depression and other mental disorders. Most collaborative care models (CCMs) in the US, for example, have incorporated patient-centered decision making processes and have been demonstrated to improve clinical outcomes such as adherence to medication, depression severity, quality of life and patient satisfaction [33]. CCMs have largely been tested in adults; however, a small number of studies have been reported in young people diagnosed with depressive disorders [34-37]. Of the three studies located, two involved client choice as part of the intervention, and one did not, as described below.

A small pilot study was conducted testing a CCM based on an intervention designed for older adults and adapted for young people 12-18 years being seen in primary care [34]. The 6-month intervention included client choice of treatment with input from caregivers, and was found to be acceptable to young people, their caregivers and physicians, and depression scores improved for the majority of participants. Two RCTs have been conducted; the first randomized participants to either 12 months of treatment as usual (TAU; predominantly prescription of SSRI medication alone) or TAU plus brief cognitive behavioural therapy (CBT; five to nine sessions), ongoing consultation with a therapist and primary care provider, and follow up contact via phone for 12 months [35]. Participants were not offered a choice in terms of medication or psychological therapy. A weak effect was found for the CCM intervention, which may have been influenced by the TAU condition being relatively effective, the small sample sizes and the low adherence to selective serotonin reuptake inhibitor (SSRI) medication in the CCM intervention group. The second RCT, the Youth Partners in Care study [36,37] designed an intervention aimed to provide young people aged 13-21 years who were being seen in primary care settings with improved access to evidence-based treatments and compared this to usual care. As part of the CCM intervention, participants were informed about, and involved in, making decisions about treatment options. Similar to results of studies with adults, the 6-month intervention significantly improved depression severity, quality of life and patient satisfaction. While there is an increased cost associated with CCMs, they may in fact be a prudent investment given their effectiveness in improving clinical outcomes and the financial costs and losses seen with untreated depression. The results from these studies offer insight into the effects of CCMs, yet it is difficult to tease apart the contribution of the patient-centered elements. Therefore, the effects seen with CCMs compared to SDM only interventions may be somewhat different.

Both SDM approaches and CCM approaches that afford participants treatment choice, assume that clients have a desire to be involved in making decisions about their own treatment and care. A narrative review investigating factors related to patient preference for involvement in decision making concluded that this was influenced by demographic variables, experiences of illness and medical care, diagnosis/health status, type of decision, patient literacy about their condition, attitude towards involvement and relationships with health providers [38]. Studies investigating preference for involvement in adults diagnosed with mental disorders have found a consistent and strong preference for involvement [39,40], and that it is feasible to do so [e.g. [41]]. Despite a desire for involvement, studies measuring levels of SDM in consultations relating to adults with mental disorders have consistently found low levels of involvement [40-45] and the authors were unable to find any studies that have measured SDM behaviours within consultations with young people diagnosed with mental disorders, nor preference for involvement in young people with mental health disorders. The context for treatment decision making for depression is likely to be different than treatment decision making in other health areas [46], and for young people even more so. Given this, there is a need to consider factors related specifically to this population.

Qualitative methods have been used to investigate specific aspects of depression care for different populations. Adult participants enrolled in a CCM intervention study were interviewed about their experience of collaborative care, and these data were used to amend and improve the model for future research projects [47]. Focus groups have been used to investigate the attitudes and preferences of different ethnic groups in relation to treatment for depression [48], as well as stigmatizing beliefs about depression and help seeking for depression [49]. These studies have highlighted the importance of stigma on help seeking behaviours and adherence to treatment. Experiences and beliefs about treatment for depression have been explored in interviews with adults [46] and, of specific relevance to this study, young people aged 14-19 years who were involved in either interviews or a focus group [50]. Both the adult and adolescent samples wanted more information about depression and the available treatment options, as well as support from their clinicians to make treatment decisions.

Given the paucity of data in the area, particularly for clients seen in tertiary mental health settings, we felt that a descriptive account of young peoples' experiences and beliefs about treatment decision making for depression would be a useful starting point from which further work into SDM and DAs for this population could build upon. We also felt that obtaining accounts from caregivers was imperative given the ages/developmental stages and the likely involvement of caregivers in the decision making processes. The aim of the current study therefore was to investigate the experiences and beliefs of young people who had been diagnosed with depressive disorders and their caregivers. Specifically, of interest was the degree to which young people and their caregivers were involved in treatment decision making, and how involved they wished to be. First and foremost, this study sought to obtain rich descriptive data on the above topics in and of themselves; however, a secondary purpose was to investigate whether or not treatment decision making could be improved at each service and to use the data to inform the development of a decision support tool.

## Methods

### Research team and reflexivity

The interviews were conducted by MS a female PhD candidate with experience in conducting qualitative, semi-structured and structured clinical research interviews with young people diagnosed with mental disorders. A relationship was established briefly with each interviewee by telephone and again in person before the interview.

### Study design

An overarching social constructionist perspective guided this project [51,52], and the methodology employed was thematic analysis [53]. The interview probes were designed to 'lead' the interviewee as little as possible, and the dialogue between the interviewer and interviewee was treated as equally relevant to the data. Ethics approval was obtained from the relevant local committee (Melbourne Health Research and Ethics Committee; reference number 2008.19). Parental or guardian consent was obtained for participants aged less than 18 years old.

### Participant selection

A purposive sample was recruited, as we wanted to obtain descriptions of experiences and beliefs from young people and caregivers who had experienced and preferred different involvement styles in relation to treatment decision making for young people. The project was presented to clinicians from each service at staff and clinical review meetings, after which clinicians were asked to provide information about the study to clients and caregivers who met the inclusion criteria. Interviews were conducted until a diverse range of experiences and views had been covered, including experiences with school based services, primary care services, and both public and private specialist mental health services. Additionally, interviews were conducted until rich descriptions of passive, shared and autonomous involvement, from a variety of clients and caregivers, both in terms of experiences of and desire for such involvement. Time was taken to review interview data as recruitment proceeded in order to ensure that the data collection ceased only once all these domains had been covered. Ten clients and five caregivers were recruited in all. Fewer caregivers were recruited because their experiences and views were more homogenous and saturation was achieved sooner.

### Inclusion Criteria

• Young people aged 12-24 years old who had received treatment for a major depressive disorder (MDD) whilst aged between 12 and 18 years old; or

• Any caregiver of a young person aged 12-18 years old, where the young person has been in receipt of treatment for MDD.

• Sufficient language skills and intellectual capacity to provide informed consent and participate and not currently experiencing a psychotic episode

### Setting

Participants were recruited from two services between October 2008 and May 2009: Orygen Youth Health (a specialist youth mental health service for young people aged 15-24 living in the north western metropolitan area of Melbourne, Australia) and headspace Barwon (an enhanced general practice service for young people aged 12-25 living in the satellite city of Geelong, 75 kms south-west of Melbourne).

### Data collection

The interview probes were based on a previously published focus group schedule [54] and modified to meet the aims of the project (see additional file 1: Interview probes). Rather than following the probes verbatim, interviewees were initially asked to describe their experiences of treatment decision making and were then afforded the opportunity to describe these experiences in their own way. The interviewer was then free to ask for clarification or to encourage participants to elaborate further on their accounts. The probes were then used at appropriate time points as the interviewees described their experiences, or to facilitate discussion if the interviewee was slow to generate discussion, and again at the end of each interview to address any topics that had not already been covered. Two additional probes were added (as seen in additional file 1: Interview probes); 'What different types of service experiences have you had?' was used as the first probe in order to encourage interviewees to initiate dialogue in their own words, and 'What constitutes *true *involvement for you?' was added in order to clarify the ways in which interviewees conceptualized involvement. The interviews were audio recorded and transcribed using an orthographic (verbatim) style, and field notes were taken during each interview. Each interview lasted between 13 and 110 minutes (mean = 43.6 minutes).

### Data analysis

Analysis was undertaken in accordance with Braun and Clarke's description of thematic analysis [53]. The analysis was theoretically driven in that main themes were decided before the interviews were conducted (and the interview probes were based on these themes, for example 'experiences of involvement in treatment decision making'), however the analysis was also inductive to an extent in that new themes were also derived from the data. More specific coding was done within each theme. Initial data coding occurred during transcription, followed by a secondary coding process conducted after all interviews had been transcribed. Themes were then compared within and across groups (clients and caregivers). Theme and coding matrices were used to organize data items and sets and to generate a thematic map. When analyzing accounts of involvement, themes were informed by Charles et al's [19] description of decision making approaches and analytical stages of decision making, as well as the distinction drawn by Elwyn and Edwards [25] between the decision-making process and who actually makes the final decision (both described above). Once analyzed, the data were summarized in a report sent to participants inviting feedback.

## Results

The key findings are summarized in Table 1. A more detailed description is given below.

**Table 1:** Summary of results from clients and caregivers related to experiences, beliefs and barriers to involvement.

	Clients	Caregivers
**Experiences: Client involvement**	• Varied according to client, clinician and service • Didn't always match preference • Most clients experienced different types of involvement • Less involved in certain settings (e.g. detoxification units, inpatient units) • Satisfaction with level of involvement varied	• Usually encouraged involvement of their offspring • Did so to promote engagement in service and personal development

**Experiences: Caregiver involvement**	• Many clients did not have caregivers involved • Clients who did not have caregivers involved described finding decision making challenging due to a lack of support • Clients who did have caregivers involved described at least one negative experience each where caregiver involvement was detrimental to decision making	• Experiences relatively homogenous • Felt involvement was usually limited to practical tasks • At times felt removed from clinical encounters, including treatment decision making • Caregivers asked for information about their child but not always given the information they wanted • Satisfaction with level of involvement varied and was influenced by characteristics of the young person and of the caregiver themselves • Many caregivers found confidentiality policies based on age problematic

**Experiences: Clinician involvement**	• All clients wanted some clinician involvement • Some clients wanted only specific clinicians involved (e.g. case manager but not doctor) • All but one client wanted clinician involvement to be of a collaborative nature • Most clients wanted to weigh up the potential risks and benefits of treatment options with clinicians	• Most caregivers wanted to trust clinicians as experts • Most caregivers wanted to be trusted as those who knew the most about their children • Caregivers reported either themselves or the clinician making the final decision • Trust in clinicians was dependant on perceived quality of care

**Experiences: Information**	• Provision of information varied across clients, clinicians, services, and also within clients across time • Information received was lacking or poor • Many clients sought information elsewhere • Some clients felt reluctant or unable to ask for more information • Information valued as important for decision-making • Clients wanted honest information about treatment options and likely outcomes to facilitate realistic expectations	• Provision of information was poor • Lack of information compounded feelings of exclusion and confusion • Some caregivers received information via their child • Some caregivers sought information elsewhere

**Beliefs: Desire for involvement**	• Desire for involvement varied both within and across clients • Most clients wanted a collaborative style • Trust, age, severity of symptoms and levels of support influenced preference for involvement • Clients distinguished between decision making process and making the final decision	• All caregivers wanted some involvement • Degree of preferred involvement varied, including preference for who makes the final decision

**Beliefs: Importance of involvement**	• Client involvement important for engagement process, adherence to treatment, safety, autonomy and empowerment • Consideration of personal characteristics, values and preferences was important to clients • Having the final say was perceived as a basic right	• Caregiver involvement important because of knowledge about offspring and continuity of care compared with limited time with clinicians • Client involvement important but extent of preference for client involvement varied

**Beliefs: Negative aspects of involvement**	• One client sited immaturity and another felt that young people were not qualified	• One caregiver felt unable to be involved when experiencing her own mental distress

**Beliefs: Improving the decision-making process**	• Suggestions influenced by experiences • Advocates on inpatient unit • Plan for therapy from the start • Wanted to be 'taken seriously' • Meaningful information that drew on existing personal knowledge • Interactive fact sheets	• Information, particularly about mental disorders

**Barriers to involvement**	• System level barriers e.g. lack of time in consultations • Relationship barriers e.g. lack of communication or trust • Personal barriers e.g. age	• Service barriers e.g. confidentiality policies • Relationship barriers e.g. exclusion by clinicians and offspring • Personal barriers e.g. own mental health issues

### Participants

Of the ten clients who participated, 5/10 (50%) were male, 8/10 (80%) had a self reported comorbid mental disorder (anxiety disorders, borderline personality disorder, substance use disorder and/or Asperger's disorder) and their ages ranged between 15 and 24 years old (mean age 20.3 years). All caregivers were female, aged between 40 and 55 years old (mean age 47.2 years), and caring for their own offspring (not necessarily the clients participating in this study).

### What currently occurs?

#### Client involvement

Experiences of involvement in the decision-making process varied across clients, as well as across different services and clinicians. Clients' experiences of involvement did not always match their preferred level of involvement. Most clients wanted some form of collaborative involvement, whereby they would be involved in the analytical stages of decision making (as described by Charles and colleagues [19]) along with their clinician. Yet it was common for the same client to experience both collaborative and paternalistic models (e.g. where involvement of clients was passive in that they were involved at a very minimal level). For example, client 01 described very passive experiences of treatment decision making, where his input and information sharing was minimal:

"the doctors used me to ascertain my medical history because I was the only one who could remember all of the drugs that I had been on, um, and that was as far as my involvement went in the process, and as for information... nuh"

Rather than feeling as if he was part of the decision-making process, he reported that he "would be sitting in the corner and they (clinician and caregiver) would be talking about me". Client 04, on the other hand, who was comfortable with researching treatment options in his own time, experienced a very collaborative approach with his psychiatrist in regards to decision making about medication. Along with open discussions between them during appointments, they also both took on tasks related to the decision-making process:

"she gave me a list of two or three different medications and said that these would probably be one of these would probably be what you'd be on... and she said go home and do some research on them if you want and tell me what you would like to be on if you do choose to go on medication"

Clients reported less involvement in decision making when treated on an inpatient unit, in detoxification units and forensic services. In these examples they described not only having decisions made for them, but also a lack of shared information exchange or deliberation [19]. One example of this was provided by client 01 who described his experiences at inpatient units as "horrible" and like being in a "dictatorship":

"Sometimes you don't even know where the decisions are coming from... they just like get made and you have to live with the consequences of those decisions... it's like, I've been in there before and like had a nurse bring a cup of medication out to me... and I wasn't taking medication the day before when I came in, all of a sudden there was this huge cup of medication in front of me. I hadn't even seen a doctor"

Some participants were accepting of this decrease in involvement, for example because they felt too unwell to be involved, whereas others were not, as in the above example.

Caregivers reported actively encouraging their sons and daughters to be involved in the decision-making process. They generally saw this as important not only for their engagement in the service, but also for their development as individuals in terms of maturation. Caregiver 03 said:

"I feel you know at fifteen and sixteen... they have to start taking some responsibility for themselves. They have to do that break away from mum bit. That's the whole teenage thing."

Two caregivers added caveats; one felt that her son should feel as if he was involved, but ultimately that she should decide what was best for him, and another felt that her son should be involved in so far as providing information and being informed, but that the clinician should make the final decision.

#### Caregiver involvement

Most caregivers had similar experiences of the ways in which they were involved both in the care of their offspring and also in the decision-making processes. Caregivers reported having essential roles in terms of practical support such as facilitating service use (e.g. driving their children to appointments) and managing medication (e.g. filling prescriptions for medication), however they reported being quite removed from treatment overall, including from both the decision-making process itself and also making decisions about treatment. All caregivers had been asked by clinicians for information about their child, in line with the passive involvement category defined by Charles and colleagues [19], but few were consulted fully about treatment decisions. Whether or not this was a concern to the caregiver differed, and this was influenced both by their appraisal of their child's capacity to engage in treatment and make decisions, and also by their level of trust in the treating clinician and/or team. For example, when caregiver 02's son was put on medication, "I wasn't asked for my opinion but that didn't worry me because I thought, these people are supposed to know what they're doing". It was also influenced by caregivers' perceptions of themselves; for example, one caregiver who was a nurse and researched medications thoroughly felt that it would be beneficial for her to be involved, whilst another caregiver who was diagnosed with Bipolar Disorder said that at times she wanted to be involved because of her knowledge based on experience, whereas at other times she couldn't be involved because she was unwell herself. Many caregivers also spoke of the difficulties they had experienced with age-based policies at services, such as caregiver 03:

"Because at the age of sixteen they're sort of classed... almost like an adult... I find that hard, because she's not an adult, she's not an adult until she's eighteen, and until that time I'm responsible for her. So if I'm responsible for her... I need to have information on what's happening within her treatment."

Caregivers reported that this lack of knowledge impacted on their ability to provide the care that they wanted.

In terms of caregiver involvement from the perspective of clients, four clients had been living in foster care or under custody orders from early ages. Any involvement from parents or other caregivers such as case workers was very limited, and at times that lack of support made decision making difficult: client 08 described feeling alone in the decision-making process because she was "making (my) own decisions bringing (myself) up". This sense of needing to be self-reliant was often spoken about in relation to clients' perception of involvement being a basic right (as discussed below in 'Importance of being involved'). Many of the clients who did have caregiver involvement reported at least one negative experience. All clients felt that caregivers should be involved to some degree, however, and there was a general consensus that caregivers should play a *supportive *role rather than a *decision-making *role.

#### Clinician involvement

When asked about the involvement of clinicians, some clients felt that it was important for clinicians to be involved in the decision-making process, others said that it depended on the clinician (e.g. one client was happy for his case manager to be involved, but didn't want his doctor involved), and nobody advocated for a model where clinicians' input was excluded. All clients wanted clinicians' input to be of a collaborative nature except for client 03, who said that he wanted the clinician to set the agenda for treatment. He felt that if it were left up to him, then he wouldn't feel confident in his choices and may miss opportunities for recovery given the time limitations of the service. An example of the desire for a collaborative approach was client 05 who wanted her clinician to provide information to her, but also for her clinician to consider her past experiences and wishes, and to follow up and monitor her in order to demonstrate that they 'care'. Interviewees were also asked directly whom they thought should weigh up the potential risks and benefits of treatment options. All responses except for two focused on the client and the clinician doing this together, or the client doing this after the clinician had explained the potential risks and benefits or offering their advice. Clients 01 and 03 also felt that caregivers should be involved in this 'weighing up' process, however client 03 qualified this by saying that it should be dependent on the age of the client and also that the information given to clients should be 'watered down' so as not to deter them from seeking help.

Most caregivers felt a need to be able to trust the experience and knowledge of the clinicians (as "experts"), whilst at the same time acknowledging that they themselves were the people who "know (their) kids, know what (they're) like" [caregiver 03]. So although some caregivers were willing to trust clinicians implicitly (even if they had reservations), others wanted clinicians to act more as providers of information and for caregivers themselves to have the final say when making decisions. Caregivers reported mixed feelings about the quality of care provided by different clinicians, and this impacted on the level of trust they felt for each clinician.

#### Information

The level of information provided to clients varied across clients, clinicians, services, and also within clients across time. Generally the information received was described as lacking or poor. Many clients sought information elsewhere, including other clinicians (e.g. pharmacist), the Internet, and asking friends and family members. One client [client 04] even attended a conference on mental health to better inform himself. Some clients felt reluctant or unable to ask for more information from their clinician, particularly if the rapport was compromised. Yet information was seen as an important factor in the decision-making process, especially in terms of feeling comfortable with the decision. The type of information desired by clients overall was summarized well by client 10, who felt that it was important to know about "alternative stuff" (treatment options), to have "realistic expectations", good information about cognitive therapy and medication, and to be provided with honest information about potential risks (mainly side effects). Caregivers reported receiving even less information, and this compounded their feelings of exclusion (e.g. lack of awareness of what was going on during clinical sessions) and confusion (e.g. coming to terms with the experiences, diagnoses and treatment options for their child). Some caregivers received information from their child who shared items such as fact sheets with them, and others initiated their own research (usually on the internet).

### What should occur?

#### Desire for involvement

The majority of clients preferred a collaborative style approach (whereby both the doctor and client worked together to make the decision about treatment), although desire for involvement varied both within and across clients. Additionally, views about who should be involved and their roles varied within the different preferences for involvement. If there was a certain level of trust in clinicians and/or caregivers (e.g. good rapport, a feeling of mutual respect), then some clients were willing for their own involvement to be less prominent. Issues that clients reported as having influenced their desired level of involvement were age, severity of symptoms and levels of support. A distinction was often drawn between being involved in the decision-making process (e.g. discussing the options) and making the final decision [25]. For example, client 02 was happy to be quite passive in the decision-making process (e.g. not be involved in information sharing or discussing the potential risks and benefits of treatment options), but he wanted to be the one to make the decision. Client 03, the only participant who thought that young people should be involved as *little *as possible in the decision-making process, also described an experience where he ceased medication without the involvement of his clinicians or caregiver in order to illustrate that the decision was ultimately his.

All caregivers wanted some involvement, although the degree to which they wanted to be involved varied. One caregiver said that she would listen to the opinions of her son and the clinicians, but then she would always make the final decision. She even went so far as to say that if the clinician didn't agree with her that she would take her son to a different service. She also reported that she often went in to her general practitioner already having made her mind up about the treatment decision outcome, and this included asking for (and subsequently receiving a prescription for) antidepressant medication for her son. Another caregiver held contrasting views and felt that clinicians should always be the ones to make decisions about treatment and she said that she just had to trust that the right decision was being made, even if she (or her son) didn't agree with the outcome. The remaining three caregivers wanted to play equal roles in collaboration with their child and the relevant clinicians.

#### Importance of being involved

All but one of the clients advocated for significant client involvement; as mentioned above, client 03 did not think that young people should be involved in decision making about treatment for depressive disorders. For others, there were a variety of reasons given about why it was important to be involved in the decision-making process. These included the engagement process, adherence to treatment, safety, autonomy and empowerment. Client 01 felt that the level of involvement "impacts (my) willingness to seek treatment" both at the time and in the future, and his experiences of not being involved made it difficult for him to want to subsequently seek help or agree with decisions. Interviewees also thought that their positive experiences of involvement had impacted on their adherence to medication. Although he generally adhered to his medication regime, client 01 felt that if he was more involved by being provided with more information, then "I probably would have been more happier to take it (antidepressant medication)". For others who had been non-adherent in the past, improvements in the decision-making process meant that they were more willing to take medication.

Feeling empowered and autonomous was important in and of itself for some clients, and for others this was also important for safety (e.g. being able to recognize side effects and knowing what to do about them). Client 10 believed that:

"young people need to feel control and they need to sort of feel empowered and I think they should be informed about... the drug and everything like that so yeah I think they should be pretty involved in making that decision to go on the medication".

Without involvement, client 05 felt like "things (are) out of my power or out of my control", but when she did experience a collaborative approach, this opened up a dialogue between the clinician and herself:

"I know I was really concerned about being safe about it too... I was able to ask questions without feeling judged about like is it okay if I take the Mirtazapine at night after I've had a few drinks you know and how does it work with alcohol and these kind of things and instead of someone saying no you shouldn't really drink when you're taking it it was more like well if you drink while you're taking it it probably doesn't have the same beneficial effects so explaining it in that way without being judging was really helpful"

Interviewees also spoke more specifically about how they wanted to be involved, and the role of personal characteristics, values and preferences was often spoken about before interviewees were asked about these topics directly. Having the final say was seen as a given, a basic or human right: "because you know they're (young people) human they should be able to make decisions" [client 10]; "doctors throw in suggestions and that but in the end no one can make me go anywhere really" [client 07]. The importance of being involved in treatment decision making also began before treatment was sought for some clients. Client 04 had two experiences of seeking help; one that involved being told by his parent that he was going to see a clinician whereby he failed to engage, and another where he was asked if he would like to "do something" about feeling depressed, after which he agreed to attend and engaged well.

All but one caregiver felt that their involvement was important because they knew their son or daughter in a different way to their clinicians. Also, their care for their child remained constant, whereas involvement with services and clinicians was more infrequent and changed over time. As caregiver 03 put it: "they see a psychologist what, once a week, once a fortnight, once a month in some cases. I'm the one doing the ongoing care." Caregivers also thought that it was important to involve the young person as well, although the extent to which they felt this should be done varied.

#### Negative aspects of being involved

When asked, only two clients could think of negative aspects of being involved in the decision-making process. Client 09 felt that some young people might be too immature to be involved and client 03 felt that "if you knew what you were doing you wouldn't be in therapy", and that for people with a "mental illness", information should be kept to a minimum. The only negative aspect of being involved from the caregivers' perspectives was reported by caregiver 02 who felt unable to be involved when she was experiencing mental distress herself (as discussed above).

#### Improving the decision-making process

Suggestions for how to improve the decision-making process from clients varied and were influenced by the experiences that each client had. For example, client 01 had negative experiences at an inpatient unit, so he felt that there should be advocates placed on the ward in order to support the decision-making choices of the client. One client (03) who had expressed concern about making the most of therapy sessions said that he would have liked a more structured plan about therapy from the beginning, because early on he "didn't really know where it was all heading". He said that without a clear plan it was "hard to come in every week sometimes when you don't know what's coming next" and now that he was nearing discharge from the outpatient service he would like to have a clearer idea about how far he had progressed in relation to where he "should be" at. For other clients, "being taken seriously" (05) as a young person was key to improving the decision-making process. As one client put it (01): "some services, like, you could be there as a patient and they would still want you to be twenty five with a bachelors' degree before they would take your opinion on anything".

The majority of clients felt that more information was needed in order to improve the decision-making process, and it was important for them that the information drew on existing personal knowledge so that it was meaningful. Client 06 provided an example of a time when she received information that was in line with her understanding of medication, and said that it was beneficial because the psychiatrist "could have just given me a chemical breakdown of the thing and that wouldn't have been helpful at all". Client 09 wanted more information in the form of fact sheets "but ones that you've gotta fill out and stuff"; that they were interactive was important to him. Client 05 felt that more information would have helped her to avoid a lot of the negative experiences she had when seeking help during her teenage years; when asked what information she would have liked, she said that it would be "amazing if I could see on a piece of paper options for treatment my god that would just be insane... that would be mind blowing to discuss what I think would work best with my personality". This response was made without prior discussion of shared decision making or decision support tools. Caregivers also felt that more information would have improved their experiences, particularly in relation to information about mental disorders.

### Barriers to involvement

#### Barriers to involvement in the decision-making process

Clients spoke about barriers to involvement in the decision-making process in three different contexts: at a system level, at a relationship level with clinicians and at a personal level. In terms of system-level barriers, a lack of time for questions during consultations was raised for clients who were treated in general practice and inpatient units. Barriers at a relationship level included miscommunication or a lack of communication with their clinicians, and breaches in trust (clients not trusting the clinician and/or clients not feeling trusted by their clinician) as issues. Perhaps surprisingly, symptoms of depression were only described by two respondents as being a barrier to being involved in the decision-making process (05 and 08), and the only other personal barrier that was reported was being young, where one client (01) likened being young to his negative experiences at an inpatient unit: "I wasn't involved then [when aged 12-13], like a lot of the times I didn't even like consult with people making the decisions it was a lot like um being an inpatient in my own life". For caregivers, the main barriers related to service settings and clinicians, although caregivers also reported instances where their child had excluded them from the decision-making process. Caregivers spoke about age ranges not necessarily matching developmental stages, and how this made it difficult to respect the confidentiality policies of services.

## Discussion

The most striking finding from these data is the variability in experiences of and desire for involvement in treatment decision making, both within and across clients, clinicians and services. Yet involvement, at some level, in the decision-making process was important to all clients for a broad range of reasons. This complements results from research into preferences for involvement in adults with mental disorders [36-38], and it would be of benefit to further investigate the preferences for involvement that young people with depressive disorders seen at a larger range of services have, to further increase our knowledge about the generalisability of this finding. Particularly given that young people [e.g. [55]] and adults diagnosed with depression [e.g. [56]] have demonstrated increased preferences for involvement. Despite showing a strong desire to be involved in the decision-making process, all clients wanted at least some involvement from their clinicians, which supports a collaborative model rather than an autonomous model [19]. It also demonstrates that client preferences for involvement do not always fit the three main models of involvement (e.g. paternalistic, shared and autonomous). Therefore, a more flexible understanding of involvement that incorporates more complex combinations of preferences is necessary [20,25]. The desire for involvement of caregivers varied across clients. Clients' accounts of what constituted true involvement for them focused on factors related to key aspects of the client-clinician relationship, such as engagement and adherence, as well as patient centered goals such as autonomy and empowerment. This supports the notion that conceptualizations of involvement should acknowledge and consider the views and feelings of clients (and caregivers) about their relationships with clinicians [21,57]. In line with recent calls promoting SDM for mental disorders [14,15], these data support a focus on involvement in decision-making processes for young people with depressive disorders.

The provision of information was also variable across clinicians and services, yet most clients and caregivers voiced a desire for more information. This is in line with previous research investigating the experiences and preferences of adults and adolescents receiving treatment for depression [46,50]. For clients it was important that this information accounted for their values and preferences. Some clients felt unable to ask for information, even though they felt that they didn't have sufficient information to be involved in the decision-making process or understand why a treatment was being offered and/or feel satisfied with the decision-making process. Significant barriers were discussed by clients, both in terms of access to services at all and also in terms of being involved in the decision-making process once gaining access to a service.

There are several reasons why informative resources that promote the inclusion of young people in decision-making processes are difficult to produce and may not be available. Reasons may include: a lack of evidence to base information on and the need to update resources according to the latest evidence; challenges with dissemination (e.g. translating evidence into readily accessible and understandable resources); and barriers to implementation (e.g. enlisting the support of organizations and clinicians). Information resources need time for ongoing development and, therefore, ongoing financial commitments from services. While potentially costly, they would provide a systematic way to ensure the opportunity of involvement of each client. Given the significant problems with help seeking in this population [7,8], there is an onus on service providers to employ tactics that maximize engagement and adherence to the chosen treatment option. Past negative treatment experiences have been highlighted elsewhere [12] as a key factor related to accepting a diagnosis of a depressive disorder and, therefore, impacting on help-seeking behaviour, which was echoed in the findings of this study. The need to choose treatments that are preference based and clinically effective in collaboration with the young person upon initial engagement in a service is twofold; with the hope that the treatment will work first time round, and if this is not achieved, that the young person will be willing to remain engaged and pursue further treatment options.

Caregivers found barriers in gaining access to services for their children, but also barriers to being involved in their care once accepted into services. The issue of the age of their children and associated confidentiality policies was the biggest concern reported by caregivers. Whilst such policies are unlikely to change, the use of decision support tools may be one way in which to either involve caregivers in the decision-making process (if so desired by the client) or communicate to caregivers the rationale for the decision made so that they can at least understand what is happening and why.

The experiences of the clients highlighted gaps in the decision-making process, and clients offered ways in which to improve such processes. Information that was interactive and meaningful was a priority for clients, as was feeling as if they were respected and taken seriously by their clinicians. This priority, in combination with clients' desires to have their personal characteristics, values and preferences considered, clearly supports the use of decision support tools and shared decision making. Given that preference for involvement is likely to change over time, having tools available to use on a repeated basis as decisions are revisited seems warranted. Understanding treatment options both for themselves and also to explain to caregivers if appropriate, was important for clients in order to navigate the complex process of seeking help and engaging with services.

There are several limitations to the current study. Although we undertook the study in order to obtain a rich description of experiences and beliefs based on a purposive sample, the small sample size minimizes the generalisability of these findings. While the participants were recruited from only two services, they had all experienced treatment decision-making at other services and therefore data were obtained for experiences at general practice, enhanced general practice, the public mental health system and private practitioners. We acknowledge, however, that there are likely to be characteristics unique to this sample that may not be present in the broader population. Given that a secondary aim was to look for ways in which decision making could be improved at each service, we wanted to recruit current clients of the services. This meant that recruitment was difficult, as the clinical needs of the young people needed to be prioritized. Efforts were made, however, to review the interview data as they were being collected to ensure that different types of experiences and beliefs were being addressed. Most young people and caregivers were interviewed as they were being discharged from the service, which meant that they could reflect on their time at the service as well as experiences at services prior to attending their current service. Another limitation is that participants were asked to recall events that they had experienced over several years. In line with our approach, however, we were interested in participants' accounts of their experiences rather than what actually happened. We feel that concepts such as involvement can be very subjective and different parties (e.g. doctor and patient) may describe an encounter such as treatment decision making in very different ways. How young people and their caregivers make sense of such encounters can help us to understand and improve aspects such as treatment decision making.

## Conclusions

This study is the first to consider the experiences and beliefs of young people and their caregivers about treatment decision making for depressive disorders. Clinical guidelines advocate for the inclusion of young people in such decision-making processes and the current study supports this.

The difficulty that clients reported getting accepted into services demonstrates that there is an onus on services to maximize efforts to engage clients once accepted. Given that clients reported a direct relationship between involvement and outcomes such as engagement, adherence and satisfaction with services, the importance of at least offering clients involvement in the decision-making process was highlighted. This is particularly true for clinicians or services that either precluded involvement or from which clients readily disengaged.

The factors that influence desire for involvement will not always be evident to clinicians and therefore involvement should be negotiated explicitly (rather than assuming the level of involvement that the client desires and/or can cope with) and repeatedly (because desire to be involved is likely to change over time). Caregiver involvement should be negotiated explicitly and on an individual basis. Caregivers should be supported with the necessary information about mental disorders and treatment options, particularly when they are responsible for key tasks outside of the clinical sessions (such as filling prescriptions and monitoring risk levels).

This study fills a gap in the knowledge about the context in which young people diagnosed with depressive disorders find themselves making treatment decisions, and provides the basis on which to build a body of work looking at the needs of such young people. Further advancement of this area, including the development of quality decision support tools to facilitate shared decision making, will open up the possibility of improved decision-making experiences for young people, which has the potential to improve key clinical outcomes for this population.

## Competing interests

The authors declare that they have no competing interests.

## Authors' contributions

MS conceived the project, conducted the interviews, analysed the data and drafted the manuscript under the supervision of SH and AJ. SH and AJ were also involved in subsequent redrafts of the manuscript. All authors read and approved the final manuscript.

## Authors' information

MS is a PhD candidate and Research Fellow, SH is a Senior Research Fellow and AJ is a Professorial Fellow at Orygen Youth Health Research Centre, Centre for Youth Mental Health, The University of Melbourne. This work was undertaken by MS in order to fulfill the requirements of the PhD.

## Pre-publication history

The pre-publication history for this paper can be accessed here:

http://www.biomedcentral.com/1471-244X/11/194/prepub

## Supplementary Material

Additional file 1 **'Interview probes'**. Interview probes. Click here for file
