# Supplementary material for: Experiences of treatment decision making for young people diagnosed with depressive disorders: a qualitative study in primary care and specialist mental health settings
Source: BMC Psychiatry. 2011 Dec 12;11:194. doi: 10.1186/1471-244X-11-194 (PMC3266645; doi:10.1186/1471-244X-11-194)
Supplement: Additional file 1 — 'Interview probes'. Interview probes. [file 1471-244X-11-194-S1.DOC]

**Additional file 1: Interview probes**

**Experiences**

- What different types of service experiences have you had?
- What types of decisions have you made/your clinicians made about treatment options for you?
- What options were presented to you?
- How were these options presented to you?
- How involved were you in making these decisions?
- How involved were your caregivers/parents?
- Were you informed of the possible risks and benefits of each treatment option?
- Have you ever disagreed about a treatment decision?

**Beliefs**

- Do you wish the decision-making process was different?
- If yes, how so?
- How important is everyone’s input into the decision-making process?
- How important are client, caregiver and clinician values?
- Who should weigh up the risks?
- Pros/cons of being involved in the decision-making process?
- Any barriers (e.g. in the system) to being involved?
- Anything that could improve decision-making process?
- What constitutes *true* involvement for you?
